# The Crosstalk between Prostate Cancer and Microbiota Inflammation: Nutraceutical Products Are Useful to Balance This Interplay?

**DOI:** 10.3390/nu12092648

**Published:** 2020-08-31

**Authors:** Felice Crocetto, Mariarosaria Boccellino, Biagio Barone, Erika Di Zazzo, Antonella Sciarra, Giovanni Galasso, Giuliana Settembre, Lucio Quagliuolo, Ciro Imbimbo, Silvia Boffo, Italo Francesco Angelillo, Marina Di Domenico

**Affiliations:** 1 Department of Neuroscience, Reproductive Sciences and Dentistry, School of Medicine, University of Naples “Federico II”, 80135 Naples, Italy; felice.crocetto@unina.it (F.C.); biagio.barone@unina.it (B.B.); ciro.imbimbo@unina.it (C.I.); 2 Department of Precision Medicine, University of Campania “Luigi Vanvitelli”, 80135 Naples, Italy; mariarosaria.boccellino@unicampania.it (M.B.); giovanni.galasso@unicampania.it (G.G.); giulianasettembre@gmail.com (G.S.); lucio.quagliuolo@unicampania.it (L.Q.); marina.didomenico@unicampania.it (M.D.D.); 3 Department of Health Science “V. Tiberio”, 86100 Campobasso, Italy; 4 Department of Translational Medical Sciences, University of Campania Luigi Vanvitelli, 80135 Naples, Italy; Antonella.sciarra@unicampania.it; 5 Sbarro Institute for Cancer Research and Molecular Medicine, Center for Biotechnology, College of Science and Technology, Temple University, Philadelphia, 19122 PA, USA; silvia.boffo@temple.edu; 6 Department of Experimental Medicine, University of Campania Luigi Vanvitelli, 80135 Naples, Italy; italofrancesco.angelillo@unicampania.it

**Keywords:** prostate cancer, microbiota, nutraceutical compounds

## Abstract

The human microbiota shows pivotal roles in urologic health and disease. Emerging studies indicate that gut and urinary microbiomes can impact several urological diseases, both benignant and malignant, acting particularly on prostate inflammation and prostate cancer. Indeed, the microbiota exerts its influence on prostate cancer initiation and/or progression mechanisms through the regulation of chronic inflammation, apoptotic processes, cytokines, and hormonal production in response to different pathogenic noxae. Additionally, therapies’ and drugs’ responses are influenced in their efficacy and tolerability by microbiota composition. Due to this complex potential interconnection between prostate cancer and microbiota, exploration and understanding of the involved relationships is pivotal to evaluate a potential therapeutic application in clinical practice. Several natural compounds, moreover, seem to have relevant effects, directly or mediated by microbiota, on urologic health, posing the human microbiota at the crossroad between prostatic inflammation and prostate cancer development. Here, we aim to analyze the most recent evidence regarding the possible crosstalk between prostate, microbiome, and inflammation.

## 1. Introduction

Prostate cancer (PCa) is the second most commonly diagnosed malignancy in men and the fifth leading cause of tumor-associated death worldwide [1].

Global estimations are approximating 800,000 new PCa cases and 300,000 deaths per year [2], and this condition poses a significant health concern in the future due to the gradual aging of the population. Genetics, family history, African descent, advanced age, diet, and environment are well-established risk factors for PCa development. However, the relevant pathways accounting for PCa development are not fully clarified [3,4,5]. The role of androgenic stimulation and the deficit of apoptosis of prostate cells are well-known explanations regarding the incidence and progression of PCa. Recent studies have also hypothesized a crucial role of microenvironment, infections, inflammation, and cytoskeletal changes induced by steroid integrating signals [6,7], influencing patients’ outcomes and the rationale for the immunological treatment of PCa [8,9,10,11,12].

Chronic inflammation is a prominent contributing factor to the benign and malignant prostatic growth; however, the potential stimulus that induces or maintains this chronic inflammation remains poorly characterized [13]. Inflammation, sex hormones, and many other factors (e.g., infections, diet, physical activity, drugs), are known to affect the microbiota. The microbiota is a complex community composed of fungi, parasites, bacteria and viruses living within the human body. Microbiota components interact with each other and with the host, impacting, eventually, the overall human health. The purpose of this study is to summarize and analyze the most recent evidence regarding the possible crosstalk among prostate, microbiota, and inflammation.

## 2. Prostate and Chronic Inflammation

**Figure 1:**
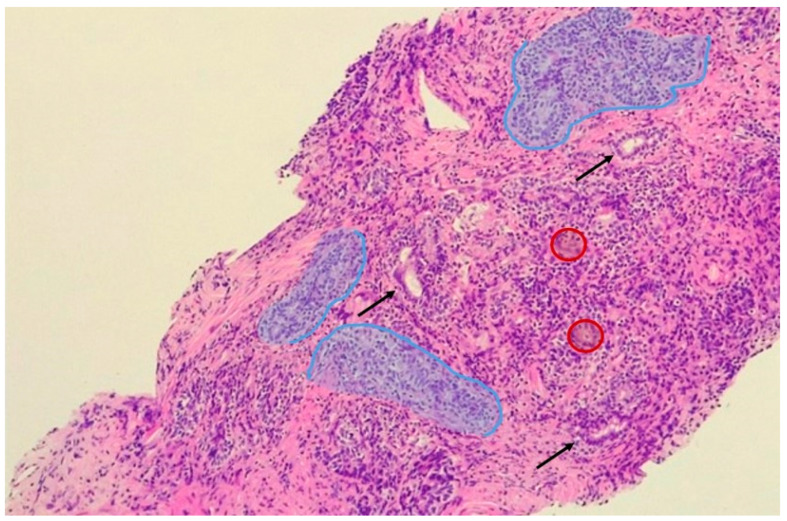
Chronic prostatitis and immune cell infiltration. Outlined in blue are aggregates of lymphocytes, plasmacells and istiocites, which surround damaged glands (black arrows). In red circles, multinucleated giant cells are outlined.

The role of inflammation in the carcinogenesis of a solid tumor is an accustomed aspect [13]. In fact, two key inflammatory cytokines, IL-6 and IL-2, have been convincingly implicated in prostate cancer pathogenesis. Inflammation may also contribute to impairing immune surveillance mechanisms, which are partially mediated by NK cells [14,15]. Repeated tissue damage and regeneration produce highly reactive nitrogen species (RNS) and oxygen species (ROS), which are responsible of cancer development and progression [16,17,18,19]. The underlying biological mechanism relies on DNA modifications of cells caused by this continuous process of damage and repair [20,21,22]. However, if there is a strong and proven connection between solid tumors and inflammation, the role of this condition in PCa development is still debatable and under revision. Different studies have suggested how chronic prostatitis could induce proliferation of stromal and glandular cells in response to ROS production, eliciting general tissue damage, and vascular injury [23,24]. ROS, moreover stimulate NF-kβ and TNF-α pathways by activating their proper kinases [25]. The morphological modification in the prostate tissue, associated with chronic and acute inflammation, is a glandular atrophy with hyperplasia called proliferative inflammatory atrophy (PIA) [26]. Up to 40% of PIA lead to the transition to a high-grade prostatic intraepithelial neoplasia (PIN), a precursor of PCa. Although some evidence of molecular changes has been observed in PIA, no certain clonal genetic alterations have been found in this condition [27]. However, genes such as NKX3.1 and CDKN1B have been shown to be downregulated in PIA, as in PIN and PCa, while the increased transcription of Hsp27 and PRDX6 could promote processes leading to tumorigenesis [28]. Several PCa susceptibility genes, such as MIC1, RNASEL, MSR1, PON1, TLR4, OGG, BRCA2 and CHEK2, are involved in prostate carcinogenesis and in other critical processes, as a host response to steroids, infection, inflammation and oxidative stress [29,30]. Furthermore, despite significant changes in inflammatory cellular infiltration between prostatitis, benign prostatic hyperplasia (BPH) and PCa have been found; the role of innate and adaptive immunity has not been completely cleared [13] (Figure 1). Chronic inflammation could have, moreover, a significant effect on cancer progression and metastatic invasion due to neo-angiogenesis and activation of epithelial–mesenchymal transitions (EMTs) [31,32]. These biologic and pathogenic processes are correlated to various molecules defined as biomarkers/indicators of normal, or pharmacologic, responses to a therapeutic intervention [33]. The control of these phenomena triggers pathways, as migration, proliferation, cell growth, apoptosis, and adhesion through various downstream effectors. The first key element that regulates cell proliferation, migration, and invasion in PCa is p85αPI 3Kinase [34,35,36,37,38]. Evidence from the literature supports the role of angiogenesis in human cancer progression, including PCa. The vascular endothelial growth factor (VEGF) is a potent angiogenic factor [39,40]. Several miRNAs, functioning as tumor suppressors or oncogenes are deregulated in prostate tumorigenesis. miRNA dysregulation progress has a key role in prostate cancer [41]. Anti-VEGF therapy and combined chemotherapy treatments trigger apoptosis in cancer and, in particular, in prostate cancer [42,43,44,45,46,47,48,49,50].

Different cancer types (i.e., lung cancer, pancreatic cancer, glioblastoma, meningioma, myeloma, and myeloma) are characterized by distinct patterns revealed by corona composition, constituting a “fingerprint” for each cancer type [51,52,53,54].

Classically, the peripheral zone of the prostate gland is a common site of PCa development, while the transitional zone is mostly affected by benign prostatic hyperplasia [55]. However, in about 20% of cases, the two conditions subsist in the same zone and, despite different pathogenic pathways, several well-established epidemiologic studies confirm that both conditions are hormone-dependent and could be associated with a previous chronic prostatic inflammation [56]. A certain degree of inflammation is almost always present when prostate specimens are sampled: the REDUCE trial demonstrated on 8224 men that, indeed, 77.6% of biopsies are positive for some grade of inflammation, with the majority (>80%) showing a mild chronic inflammation [57]. To further support these findings, men diagnosed with prostatitis have an increased risk of developing, in the future, PCa compared to those without any grade of prostate inflammation. Specifically, 18% of those patients will develop PCa [58]. Chronic and acute inflammation is also frequently found in prostate tumor specimens obtained from prostatectomies and transurethral resections [59]. A study conducted by Daniels et al. on 5821 men >65 years old reported a positive association between a previous history of prostatitis and PCa (OR 5.4, 95% CI = 4.4–6.6) [60]. Similarly, Cheng et al. showed that protracted prostatitis symptoms could significantly increase the odds of PCa in 68,675 men (RR 1.3, 95% CI = 1.10–1.54) [61]. In addition, Dennis et al. reported, in a meta-analysis of 11 case-control studies, the evidence of a statistically significant risk of developing PCa in patients with a previous history of prostatitis (OR 1.6, 95% CI = 1–2.4) [62] and analogous results were found by a similar meta-analysis on 20 case-control studies (OR 1.50, 95% CI 1.39–1.62) [63]. Finally, a recent and wide meta-analysis by Perletti et al. reported, in 422,943 patients, a significant association between PCa and previous prostatitis (OR 1.83, 95% CI = 1.43–2.35) [4]. However, despite those data, the real impact of chronic inflammation on prostate carcinogenesis has been challenging to define. In particular, it is not easy to estimate the real incidence of prostatitis due to the asymptomatic majority of cases (5–10%) [64]. Moreover, evidence that seems to show an increased risk for acute prostatitis rather than for chronic prostatitis, is influenced by the same potential detection bias.

### Etiology of Prostate Chronic Inflammation

The etiology of chronic inflammation preceding PCa development remains unknown, however, infections and chemical trauma are often correlated to chronic inflammation.

Several putative etiological agents have been identified, from the xenotropic murine leukemia virus related-virus XMRV to different strains of bacteria [58,65,66,67]. Several studies support the potential role of infectious agents in PCa etiology with evidence that up to 87% of PCa patients show microbial DNA in their prostate [68,69]. However, if no clear association had been shown with HPV or other sexually transmitted viruses, men with previous gonorrhea or syphilis infections had a 60% increased risk of developing PCa [70]. A study based on animal models reported a mutagenic activity of inflammation caused by *Escherichia coli* in the prostatic gland, with the induction of epithelial hyperplasia, an increased tendency to apoptosis, and somatic mutations [71]. Moreover, the presence of an induced prostatic infection with *Escherichia coli*, in addition to the consumption of a diet enriched with a cyclic amine, the 2-amino-1-methyl-6-phenylimidazo [4,5-b]pyridine (PhIP) (a well-known prostatic carcinogen in rodents), further increased the risk of PCa development in mice with a marked drop in survival rate compared with PhIP-alone-treated animals, thus suggesting chronic inflammation as an enabling characteristic of PCa [72]. Cai et al., reported a significant increase in Gram-positive strains in patients with chronic prostatitis and a successively diagnosed PCa [73], while other significant associations between cancer development and infection were shown also for *Mycoplasma hominis* [74] and *Trichomonas vaginalis* [75,76]. In particular, previous *Trichomonas vaginalis* infection could create a favorable microenvironment, promoting PCa cell proliferation and invasiveness (activating the epithelial–mesenchymal transition), in addition to an increased overall inflammatory state of the gland [77]. Twu et al. reported, in fact, how *Trichomonas vaginalis* secretes a protein (TvMIF), which is 47% similar to the human macrophage migration inhibitory factor (HuMIF), which is reported to be elevated in PCa [78]. *Propionibacterium acnes*, which is frequently isolated in prostate tissue, has also been thought to have an influence on the development of PCa due to the association with reported histological inflammation in prostate-derived tissue models and prostatectomy specimens [79]. To further outline the role of *Propionibacterium acnes* in prostate carcinogenesis, Ugge et al. retrospectively analyzed the association between the presence of acne vulgaris during adolescence and the occurrence of PCa in 243,187 men for a median follow up of 36.7 years; 1633 of those patients developed PCa, reporting an adjusted OR of 1.43 (95% CI = 1.06–1.92) [80]. However, a recent meta-analysis by Zhang et al. did not find a significant association between acne and PCa, questioning this relation [81]. An EPICAP study reported instead the association between sexually transmitted and urinary tract infections and PCa, with an increased risk of developing this malignancy in patients with a previous history of prostatitis (OR 2.95, 95% CI = 1.26–6.92) and in patients who did not assume non-steroidal anti-inflammatory drugs (OR 2.00, 95% CI = 1.37–2.91) [82]. To further support the role of chronic inflammation in increased PCa risk, St. Hill et al. showed how EBV, HIV, HBV, HCV, or HSV chronic infections were associated with an increased risk of occurrence of PCA. Similarly, the risk was also increased in men with other chronic inflammatory diseases or conditions such as osteoporosis, diabetes mellitus, arthritis, or cardiovascular disease; however, currently, no inflammation marker could be associated with a higher risk of PCa development [83].

## 3. Microbiota in Urological Disease

### 3.1. Urinary and Prostate Microbiota

Commensal microorganisms colonize barrier surfaces of all multicellular organisms, coevolving and adapting with the host for more than 500 million years. As result, the commensal microbiota affects many processes of their hosts via biologically active molecules, playing critical roles in human diseases, in particular cancers and autoimmune conditions, influencing the innate and adaptive immune response [84]. The discovery of communities of bacteria in the genitourinary tract and their role in urologic diseases has introduced novel factors and implications in the pathophysiology of these conditions. The advent of such molecular-based methods as the quantitative real-time PCR and amplification of 16S rRNA for the identification and characterization of microbial populations has permitted the discovery of previously unrevealed microbial populations. Historically, the bladder, and generally the urinary tract, has always been considered sterile, however, recent studies have revealed important evidence of the presence of microbes in bladders of patients without clinical infection [85,86]. Human urinary microbiota characteristics depend on the age, gender, and disease status of individuals [87,88,89,90,91,92], and understanding its role in urological diseases is of particular interest. Moreover, novel molecular methods have made it possible to characterize the bladder microbiota formed by *Burkholderia cenocepacia* and different strains of *Lactobacilli* in urologic chronic pelvic pain syndrome (UCPSS), which was considered to be defined as “the absence of identifiable bacterial infection” [93]. Particularly interesting is that, in the same condition, an increased rate of *Lactobacilli*, compared to the remaining flora, was instead revealed in the urine of patients with Interstitial Cystitis (IC) [94]. Furthermore, *Lactobacillus casei* and *Lactobacillus rhamnosus* could also have interesting applications in the treatment of bladder cancer, demonstrating a decreasing effect on rates of metastasis and recurrence due to an enhanced recruiting of natural killer cells, both in vitro and in vivo [95]. Accordingly, different studies have hypothesized a link between prostate microbiota and pro-inflammatory bacterial species. In 2016, Mandar et al. reported a lower rate of *Lactobacilli* in patients with chronic prostatitis, while Shoskes et al. reported, for the same condition, higher rates of *Clostridia* and *Bacteroides* compared with controls [96,97]. In 2015, Yu et al. described how bacterial strains present in prostatic secretions, seminal fluid and voided urine are different among patients with BPH and PCa. Specifically, there are lower rates of *Eubacterium* and *Defluviicoccus* and higher rates of *Bacteroidetes* in patients with PCa, hypothesizing the role of certain bacteria in the induction of chronic inflammatory states, with enhanced production of factors favoring tumorigenesis [98]. An analogous study conducted on 135 PCa patients by Shrestha et al. in 2018, reported an increased presence of *Anaerococcus lactolyticus* and *obesiensis*, *Streptococcus anginosus*, *Varibaculum cambriense*, *Actinobaculum schaalii*, and *Propionimicrobium lymphophilum*. All patients were previously diagnosed with urinary tract infection caused by *Enterobacteriaceae*, which were instead more abundant in patients with BPH [99]. Similar conclusions were reported by Alanee et al., confirming the possible association between urinary and fecal microbiota with PCa after examination of prostate biopsies, which were characterized by higher rates of *Streptococcus anginosus*, *Anaerococcus lactolyticus*, and *Varibaculum cambriense* [100]. Analogously, Bhudia et al., reported increased rates of *Staphylococcus epidermidis*, *Streptococci*, *Corynebacterium amycolatum*, *Peptoniphilus harei*, and *Fusobacterium nucleatum* in prostate secretions of PCa patients [101]. Cavarretta et al. reported an abundance of *Propionibacterium* spp. and *Staphylococcus* spp. in 16 tumoral and peritumoral prostatectomy specimens [102]. Similarly, Feng et al. examined 65 radical prostatectomy specimens, reporting an increased rate of *Escherichia coli*, *Propionibacterium acnes*, *Pseudomonas* spp. and *Acinetobacter*; in particular, *Pseudomonas* has a gene expression profile that strongly correlates with human small RNA’s profile, and that could be also related to metastasis [68]. Moreover, the same authors identified increased bacterial content (especially *Escherichia* spp. and *Acidovorax* spp.) in prostate specimens of African men, which were also associated with elevated tumor hypermutation, suggesting the possibility of a bacterially driven oncogenic transformation [69].

### 3.2. Gut Microbiota

The role of microbiota in urological diseases and PCa is, however, not limited to bacteria related to the urinary tract. Modification of the gut microbiota could modify the risk of incurring PCa and be influenced by the tumorigenesis process itself [103] (Table 1). Liss et al. reported significant differences in bacteria obtained via rectal swab between PCa and healthy patients, with an increase in certain genera such as *Bacteroides* and *Streptococci* and impoverishment of bacteria related to folate and biotin production [104]. Golombos et al., similarly, confirmed the abundance of *Bacteroides* in PCa patients and reported an increased presence of *Faecalibacterium prausnitzii* and *Eubacterium rectalie* in BPH patients [105]. Potential alterations of gut microbiota could influence, both directly and indirectly, prostate health via bacterial metabolites, and influence the enteric endocrine system [106]. Multiple studies have shown that gut microbiota also modulates the response to chemotherapy acting on the translocation, immunomodulation, metabolism, and enzymatic degradation of drugs [107]. This consideration, moreover, is valid also for androgen axis-targeted therapy in PCa treatment, which is influenced in its clinical response and antitumoral efficiency by gut microbiota. Conversely, androgen axis-targeted therapy enhances *Bacteroides* and *Streptococci* rates in the gastrointestinal tract while lowering overall bacterial diversity [108]. Besides, an analysis of the fecal microbiota of healthy volunteers and PCa patients by 16S rDNAsequencing, showed a greater abundance of *Akkermansia muciniphila* and *Ruminococcaceae* spp. in the microbioma of patients treated with oral androgen receptor axis-targeted therapies such as enzalutamide, bicalutamide and abiraterone acetate [109]. Finally, there are suggestions that butyrate, an anti-inflammatory micronutrient produced by *Faecalibacterium prausnitzii* and *Eubacterium rectale*, could be implicated in one of the pathways for the prevention of PCa, although further studies are required [105].

## 4. Nutraceutical Aspects in the Interplay between Prostate and Microbiota

### 4.1. Unsaturated Fatty Acids

Olive oil and unsaturated fats, high vegetable consumption, fruit intake, and allium vegetables, typical aspects of the Mediterranean diet, were related to a decreased risk of several cancer types. In particular, countries following the Mediterranean Diet have lower PCa incidence and mortality compared to other European regions. However, there are few studies that have assessed the effect of the Mediterranean diet on PCa incidence. Further large-scale studies are required to clarify the effect of the Mediterranean diet in order to establish the role of this diet in the PCa prevention [110,111]. PCa has a well-known association with food and, in particular, with fat intake; moreover, there is a relationship between PCa and gut microbiota that changes based on the diet [112]. A low-fat diet and/or intensive exercise involves changes in serum hormones and growth factors in vivo, which could reduce growth and induce apoptosis of LNCaP prostate tumor cells in vitro [113]. Low-fat diet-fed mice show significantly lower levels of prostate-specific serum antigen (PSA), insulin and Igf1 mRNA levels compared to mice with a high-fat diet, as well as a delayed tumor-growth rate in LAPC4 xenografts [114]. A high-fat diet induces, in fact, lipid accumulation in PCa and promotes metastasis via abnormal sterol regulatory element-binding protein (SREBP)-dependent lipid metabolism [115]. Several epidemiological studies suggest that an increased intake of saturated fatty acids and a sedentary lifestyle decreases the survival rate of PCa patients, whilst unsaturated fatty acids and physical activity reduce the risk of PCa [116,117]. In recent years, *n*-3 fatty acids, eicosapentaenoic acid (EPA) and docosahexaenoic acid (DHA), present in fish oil, have been found to influence cancer cell proliferation. EPA and DHA were, moreover, effective in decreasing the proliferation, invasion, and migration of prostate PC3 cancer cells as well [118]. As known, sex hormones also play an important role in the development and progression of PCa. In prostate-specific Pten-/-mice, the reduction in serum cholesterol lowers intraprostatic androgens and suppresses tumor progression, although it does not change the incidence of PCa [119]. In transgenic mice, the consumption of high amounts of unsaturated fatty acid ω-3, produces a significant slow-down of prostate tumorigenesis by affecting estradiol, testosterone, and androgen receptor levels, suggesting a specific role of unsaturated fatty acids in the regulation of sex hormones, which may be the basis of fat-induced PCa progression [120].

### 4.2. Carnitine

Carnitine, and in particular its acetylated derivative, Acetyl-l-Carnitine (ALCAR) is involved in mitochondrial membrane trafficking in catabolic and anabolic pathways. Several studies have documented the antioxidant and scavenger activity of this compound, utilized in clinical settings related to disorders where the oxidative stress acts as a promoting factor (e.g., diabetes, Alzheimer’s disease, and other neurometabolic disorders) [121,122]. ALCAR reduces PCa cell viability and induces apoptosis; moreover, ALCAR impairs the adhesion, invasion and migration of PC3, DU145, LNCaP, and BPH cells, eliciting a decreasing effect on TNF-α and other proinflammatory cytokines, such as IL-6, CCL2 and CXCL12 [123]. Besides, ALCAR was able to limit inflammatory angiogenesis, in vitro and in vivo, downregulating the VEGF/VEGFR2, CXCL12/CXCR4, and FAK pathways [124].

### 4.3. N-acetylcysteine (NAC)

*N*-acetylcysteine (NAC) is an exogenous antioxidant primarily used as a mucolytic agent and as an antidote of acetaminophen toxicity. Its effects on increasing glutathione levels and scavenging free radicals pose NAC as a powerful antioxidant. The association of NAC with phenethyl isothiocyanate (PEITC) and sulforaphane (SFN), two compounds present in cruciferous vegetables (cauliflower, cabbage, and broccoli) inhibit LNCaP and DU145 cell growth in a dose-dependent manner, increasing p21, a potent inhibitor of cyclin-dependent kinases mediating cell replication, up to apoptosis. Besides, SFN-NAC reduces PSA and the expression of the androgen receptor [125,126]. NAC alone inhibits the growth of PC3 cells suppressing the transcription of nuclear factor (NF)-κB, while increasing Cyr61 levels and activating the Erk pathway [127]. Finally, NAC shows a significant anti-migration and anti-invasion activity on DU145 and PC3 cells, limiting the metastatic ability of those cells [128].

### 4.4. Monoterpenes

Terpenoids are natural constituents of plants and animals. The most common form occurs as monoterpenes, components of essential oils of herbs and spices. D-Limonene, the most abundant monoterpene present in orange, lemon, and peppermint essential oil, has been shown to inhibit PCa cell growth via Erk pathway activation and the induction of WAF1 and p21 [129]. Geraniol, another monoterpene found in geranium and citronella plants, inhibits tumor cell growth via the induction of apoptosis in PC3 cells, activating caspase-3, reducing Bcl-2 expression and increasing Bax and BNIP3 levels. Besides, geraniol has been found to inhibit AKT-mTOR signaling without influencing mitogen-activated protein kinase (MAPK) activity [130]. A thyme honey component, the trihydroxy ketone E-4-(1,2,4-trihydroxy-2,6,6-trimethylcyclohexyl)-but-3-en-2-one exerted significant apoptotic activity in PC3 cells, through a reduction in NF-κB activity and IL-6 secretion [131].

### 4.5. Polyphenols

Polyphenols are widely studied for their beneficial effects on human health, particularly in cancer prevention. Several studies associate, in particular, catechin and isoflavone with beneficial effects on PCa. The epigallocatechin-3-gallate (EGCG), the most common catechin in green tea (>50% of the total polyphenol content), shows a great physiological activity: EGCG arrests cell growth in the G0/G1-phase and induces apoptosis in both androgen-sensitive and insensitive human PCa cells [132]. Moreover, EGCG, in both androgen-sensitive and insensitive human PCa cells, attenuated the effects of arachidonic acid (AA) in increasing cell growth and prostaglandin E2 levels by reducing the concentration of the enzyme cyclooxygenase 2 (COX-2) [133]. EGCG also acts through different mechanisms in order to arrest cell cycle and induce apoptosis, in fact in 12-week-old TRAMP mice, contrary to 28-week-old mice, it suppressed PCa development at an early stage after oral intake of EGCG by regulating IGF-1-related signaling and COX-2 levels [134]. Green tea has, therefore, an inhibitory effect on PCa tumorigenesis when assumed in large quantities. Kurahashi et al. examined the relationship among green tea consumption and PCa risk, in a large-scale prospective study of 49,920 Japanese men, reporting how subjects who drank five or more cups of green tea each day had a lower risk of advanced PCa than those who drank less than one cup per day (RR 0.52, 95% CI = 0.28–0.96) [135]. More recently, a meta-analysis on ten large studies on the incidence of green tea and PCa has shown how the risk of PCa decreases in a dose-dependent manner, with a significant reduction in the risk for subjects who drank more than seven cups a day (RR 0.81, 95% CI = 0.67–0.97 for 7 cups/day; RR 0.74, 95% CI = 0.59–0.93 for 9 cups/day; RR 0.56, 95% CI = 0.35–0.92 for 15 cups/day) [136]. Isoflavones also play an important role in the prevention of PCa, with a reduction in PCa risk related to the intake of soy isoflavone [137]. Soy isoflavones, having a structure similar to 17β-estradiol, can bind to the estrogen receptor (ER), behaving as phytoestrogens with a binding affinity and transcriptional activity stronger on ER-β than on ER-α and thus having more likely estrogenic effects in prostate tissue, which expresses higher levels of ER-β. Genistein, another isoflavone contained in fava beans, soy, and coffee, induces apoptosis of PC3 cells by suppressing NF-κB via the AKT signaling pathway [138]. In DU145 cells, genistein, EGCG, and Silymarin, a flavonolignan contained in Cardus marianus, induced the inhibition of erbB1 membrane receptor activation caused by TGFα, provoking a dose-dependent inhibition of cell growth [139]. In addition, EGCG could induce apoptosis in LNCaP cells by two pathways: the first acted on the stabilization of tumor suppressor gene p53 and on the reduction in MDM2 protein expression; the second was related to the negative regulation of NF-κB activity, leading to a decreased expression of the anti-apoptotic protein Bcl-2 [140]. In TRAMP mice, food genistein reduced PCa development in a dose-dependent manner [141]. Parallel studies in TRAMP-FVB mice showed that a low-dose genistein diet (250 mg/kg) promoted PCa growth and metastasis compared to control and a high-dose genistein diet (1000 mg/kg), showing a biphasic effect of isoflavones on PCa [142]. Paller et al. found that an increase in quercetin intake, another well-known isoflavone contained in capers, leads to a reduced risk of PCa, in African-Americans with vitamin D deficiency, while Sun et al. showed that its use, associated with metformin, inhibits the growth, migration, and invasion on PC3 and LNCaP cells by inhibiting the VEGF/AKT/PI3K signaling pathway [143,144]. Similarly, fisetin has been suggested to act as a dual inhibitor on PI3K/AKT and mTOR metabolic pathways in PCa cell lines. In addition, this compound could be used, alone or as an adjunctive drug in the chemotherapeutic treatment of PCa [145]. In two different prostate cancer cell lines, androgen-sensitive (LNCaP) and androgen-independent (DU145), cyanidin-3-O-beta-glucopyranoside (C3G), the most abundant anthocyanin in the diet, produced anti-proliferative effects through the activation of caspase-3 and the induction of p21 protein expression. Besides, treatment with C3G increased the levels of tumor suppressor P75 NGFR, indicating a possible role of C3G in the acquisition of a normal-like cell phenotype. C3G may, therefore, be considered a new therapeutic agent with both anti-proliferative and pro-differentiation properties [146]. The DU-145 cells treatment with anthocyanins extracted from black soybean provoked a significant increase in apoptosis and a significant decrease in p53, Bcl-2 and AR expressions with, in addition, a further decrease in PSA levels. Moreover, the anthocyanin treatment showed a significant inhibition of tumor growth in xenograft models [147]. Gallic acid (GA) induced apoptosis in DU145 and 22Rv1 cell lines, demonstrating, in nude mice fed with GA, inhibition of tumor growth [148]. In addition, GA reduces survival, proliferation, and invasion in PC3 cells [149]. Gallotannins, polymers formed by the esterification of GA, produce an apoptotic effect in DU145 and PC3 cell lines by decreasing the expression of different genes, such as Mcl-1, and inhibiting caspase activation [150]. Similarly, the ellagitannins of the pomegranate, named punicalagin (PN), have elicited the induction of apoptosis in PC-3 and LNCaP cells [151]. In the pomegranate, as well as juice, extract, or oil, in addition to the ellagitannins, there are also large quantities of anthocyanins that have powerful antioxidant and anticancer activities in different tumors, including PCa [152]. Caffeic acid and its natural ester-caffeic acid phenethyl ester (CAPE) are potent inhibitors of the androgen-dependent PCa lines [153]. Caffeic acid and CAPE from bee propolis showed a synergistic effect with chemotherapeutics and radiotherapy, repressing, moreover, tumor growth and AKT signals in human PCa cells [154]. Esters of cinnamic acid induce apoptosis and inhibit the growth of prostate and breast cancer [155]. Chlorogenic acid inhibits benign prostatic hyperplasia growth, probably via the inhibition of 5αR, in the animal model [156]. Ferulic acid induced the arrest of cell cycle in PC3 cells while, in LNCaP cells, it provoked apoptosis [157]. Resveratrol treatment of LNCaP cells led to the phosphorylation and the nuclear translocation of ERK1/2 (mitogen-activated protein kinase) and the accumulation of nuclear COX-2, and subsequently to the complex formation with pERK1/2 and p53 [158]. In addition, curcumin, a polyphenolic molecule extracted from the rhizome of the plant *Curcuma longa*, inhibits the proliferation of androgen-dependent and androgen-independent prostate cell lines [159]. Curcumin increases, in fact, the sensitivity of PCa cell cultures to gamma-radiation, reduces the trans-activation and the expression of AR (acting also as its antagonist), reduces the expression of EGF receptors, induces the degradation of HER2, reduces angiogenesis in vivo and the expression of VEGF [160]. Curcumin acts, moreover, as an inhibitor of the tumor necrosis factor (TNF-α) and prostaglandin E2 (PGE2) production, but increases the caspase activity (3, 8, 9) in HL-60 PCa [161]. A recent study by Chen et al. examined the anti-carcinoma potential of curcumin, treating PC3 and DU145 cells with a series of curcumin analogs of the second generation, in concentrations of 0–10 μM, founding the ability of curcumin to decrease the expression of NF-kB, mTOR (mammalian target of rapamycin), AKT and p-AKT [162]. Colonic metabolites may participate in the chemoprevention of PCa by varied polyphenol-rich diet or composite polyphenol preparations. The gut microbiota-derived metabolites of ellagitannins and green tea catechins, urolithin A (uroA) and 5-(3′,4′,5′-trihydroxyphenyl)-γ-valerolactone (M4), respectively, are, in fact, the main compounds absorbed by the human system and derived from the metabolism of these polyphenols. Stanisławska et al. established the effects of M4, uroA, and their combinations on LNCaP cells: M4 showed modest antiproliferative activity in LNCaP cells (IC50 = 117 µM; CI: 81–154), while uroA decreased proliferation (IC50 = 32.7 µM; CI: 24.3–41.1) and induced apoptosis in the same line of cells with, furthermore, a synergistic antiproliferative activity of M4 plus uroA. Besides, M4 potentiated the inhibition of PSA secretion and enhanced AR retention in cytoplasm caused by uroA [163]. Urolithins induced apoptosis in LNCaP cells, negatively influencing the levels of Bcl-2 protein and probably decreasing the expression of AR and the PSA synthesis [164]. Moreover, the gut microbiota itself is influenced by those colonic metabolites, eliciting beneficial effects on intestinal probiotic bacteria [165]. The dietary pattern has, indeed, an important and direct influence on gut bacteria composition [166]. The western diet, consisting of high-fat content and high sugar content, reduces the diversity of the gut microbiota in mice, increasing in *Bacteroides* spp. and *Ruminococcus torques* [167] while, in humans, increasing *Enterobacteriaceae* rates and significantly decreasing short-chain fatty acids in feces, one of the metabolites generated by bacteria [168].

## 5. Conclusions

Although the prostate is not an organ directly affected by gut microbiota, a wealth of evidence suggests an indirect influence of cytokines and immune changes derived by different bacterial metabolites and gut microbiota modifications. The previously reported studies support a potential role of diet and nutrition in PCa pathogenesis, partially mediated by the gut microbiota itself. The gut microbiota could be targeted to improve therapies while attenuating adverse reactions. The influence of diet and nutrients on PCa pathogenesis and progression have received increasing attention. Several animal studies have reported how certain nutrients, including fat and polyphenols, are indeed involved through a variety of mechanisms, which include inflammation, antioxidant activity, and influence on sex hormones (Table 2). Generally, a healthy dietary pattern (e.g., low in meat and high in vegetables) could help in the prevention of PCa and lifestyle-related diseases. Due to such considerations, the close relationship between gut microbiota and cancer is a research area that is receiving considerable attention. Based on recent findings, gut microbiota alterations, which are caused by various external factors such as dietary composition, are involved in all stages of cancer, including initiation, progression, treatment outcomes, and adverse reactions [169]. The mechanism by which gut microbiota may influence PCa has not been elucidated. Therefore, it is challenging to understand how microbiota and host influence each other. It could be speculated that while microbiota could affect the natural cancer history, cancer itself could change the microbiota composition. However, it is undeniable that colonic metabolites may contribute to the chemoprevention of PCa by varied polyphenol-rich diet or composite polyphenol preparations. Understanding the specifics of gut microbiota in the context of PCa is needed in the era of precision medicine for the development of personalized treatments. However, a further investigation and understanding of the relationships between microbiota and PCa pathogenesis, development, and progression are warranted.

## Figures and Tables

**Table 1:** Summary of bacteria increased in prostate diseases.

Bacterium	Localization	Findings	References
***Burkholderia cenopacia***	Bladder	Increased in UCPSS	[93]
***Lactobacillus casei/rhamnosus***	Bladder/Prostate	Increased in IC. Enhanced the recruitment of natural killer cells. Decreased in chronic prostatitis	[94,95,96]
***Clostridia* spp.**	Prostate	Increased in chronic prostatitis	[97]
***Anaerococcus lactolyticus/obesiensis***	Prostate	Increased in PCa	[99,100]
***Actinobaculum schaali***	Prostate	Increased in PCa	[99]
***Varibaculum cambriense***	Prostate	Increased in PCa	[99,100]
***Propionimicrobium lymphophilum***	Prostate	Increased in PCa	[99]
***Enterobacteriaceae***	Prostate	Increased in BPH	[99]
***Propionibacterium acnes***	Prostate	Increased in PCa	[39,102]
***Escherichia coli***	Prostate	Increased in PCa. Associated with elevated tumour hypermutation	[39]
***Pseudomonas* spp.**	Prostate	Increased in PCa. Expression profile related to metastasis	[39]
***Acinetobacter***	Prostate	Increased in PCa	[39]
***Acidovorax***	Prostate	Increased in PCa. Associated with elevated tumour hypermutation	[39]
***Bacteroides* spp.**	Prostate/Gut	Increased in chronic prostatitis. Increased in the gut in PCa. Further increased in ADT.	[97,104,105,108]
***Staphylococcus epidermidis***	Prostate/Prostatic secretions	Increased in PCa	[101,102]
***Streptococcus anginosus***	Prostate/Prostatic secretions/Gut	Increased in PCa. Increased in the gut in PCa	[99,100,101,104,108]
***Corynebacterium amycolatum***	Prostatic secretions	Increased in PCa	[101]
***Peptoniphilus harei***	Prostatic secretions	Increased in PCa	[101]
***Fusobacterium nucleatum***	Prostatic secretions	Increased in PCa	[101]
***Bacteroidetes* spp.**	Prostatic secretions	Increased in PCa	[98]
***Defluviicoccus***	Prostatic secretions	Decreased inPCa	[98]
***Eubacterium rectalie***	Prostatic secretion/Gut	Decreased in PCa. Increased in the gut in BPH. Could prevent PCa via increasing butyrate	[98,105]
***Faecalibacterium prausnitzii***	Gut	Increased in BPH. Could prevent PCa via increasing butyrate	[105]

Abbreviations: PCa (prostate cancer), BPH (benign prostatic hyperplasia), ADT (androgen deprivation therapy), UCPSS (urologic chronic pelvic pain syndrome), IC (interstitial cystitis).

**Table 2:** Summary of the effects of several natural compounds.

Substance	Source	Findings	References
**Eicosapentaenoic acid** (**EPA**)	Fish oil	Decreases proliferation, invasion and migration of PC3 cells.	[118]
**Docosahexaenoic acid** (**DHA**)	Fish oil	Decreases proliferation, invasion and migration of PC3 cells.	[118]
**Acetyl L-Carnitine** (**ALCAR**)	Meat, tempeh, cod	Induces apoptosis and impairs migration and invasion of PC3, DU145, LNCaP cells decreasing TNF-α, IL-6, CCL2, CXCL12. Limits angiogenesis downregulating VEGF and FAK.	[123,124]
***N*-Acetylcysteine** (**NAC**)	Allium plants	Inhibits invasion and migration of DU145 and PC3 cells.	[128]
***N*-Acetyl-S-**(***N*′-phenethylthiocarbamoyl**)**-l-cysteine** (**PEIT-NAC**)	Cauliflower, cabbage and broccoli	Inhibits LNCaP and DU145, increasing p21.	[125,126]
**dl-Sulforaphane *N*-acetyl-l-cysteine** (**SFN-NAC**)	Cauliflower, cabbage and broccoli	Inhibits LNCaP and DU145 cells, increasing p21. Reduces AR and PSA.	[125,126]
**D-Limonene**	Essential oil of orange, lemon, peppermint	Inhibits PCa cells, activating ERK and inducing WAF1 and p21.	[129]
**Geraniol**	Essential oil of geranium and citronella	Induces apoptosis of PC3 cells, activating caspase-3, reducing bcl-2 and increasing Bax and BNIP3. Inhibits AKT-mTOR.	[130]
**Trihydroxy ketone E-4-**(**1,2,4-trihydroxy-2,6,6-trimethylcyclohexyl**)**-but-3-en-2-one**	Thyme honey	Induces apoptosis of PC3 cells via reduction in NF-κB and IL-6.	[131]
**Epigallocatechin-3-gallate** (**EGCG**)	Green tea	Induces apoptosis of PCa cells, reduces COX-2, regulates IGF-1. Inhibits via erbB1 DU145 cells growth. Induces apoptosis of LNCaP, stabilizing p53, reducing MDM2 and downregulating NF-κB.	[132,133,139,140]
**Soy Isoflavone**	Soy	Binds ERs, with a stronger activity on ER-β.	[137]
**Genistein**	Fava beans, soy, coffee	Induces apoptosis of PC3 cells through the suppression of NF-κB via AKT. Inhibits via erbB1 DU145 cells growth.	[138,139]
**Silymarin**	Cardus marianus	Inhibits via erbB1 DU145 cells growth.	[139]
**Quercetin**	Capers	Reduces PCa risk in vit.D deficiency, inhibits growth, migration and invasion of PC3 and LNCaP cells, inhibiting VEGF, AKT, PI3K in combination with metformin.	[143,144]
**Fisetin**	Strawberries, apples, onions	Inhibits PI3K, AKT and mTOR in PCa cells.	[145]
**Cianidina-3-O-beta-glucopiranoside** (**C3G**)		Inhibits proliferation of LNCaP and DU145 cells trough activation of caspase-3 and induction of p21. Increases P75NGFR.	[146]
**Gallic acid** (**GA**)	Gallnuts, sumac, tea	Induces apoptosis of DU145 and 22Rv1 cells. Reduces proliferation of PC3 cells. Its polymers (gallotannins) decrease Mcl-1.	[148,149,150]
**Punicalagin** (**PN**)	Pomegranate	Induces apoptosis of PC3 and LNCaP cells.	[151]
**Caffeic acid phenethyl ester** (**CAPE**)	Bee propolis	Reduces AKT in PCa cells.	[154]
**Ferulic acid**	Cereals	Causes cell cycle arrest in PC3 cells while inducing apoptosis in LNCaP cells.	[157]
**Resveratrol**	Grapes, bluberries	Inhibits LNCaP cells via ERK1/2 and inducing p53.	[158]
**Curcumin**	Curcuma longa	Reduces AR, EGF, VEGF while inhibiting TNF-α and PGE2 in PCa cells. Inhibits NF-kB, mTOR, AKT and p-AKT expression.	[160,161,162]
